# Bio-efficacy and wash resistance of MAGNet long-lasting insecticidal net against wild populations of *Anopheles funestus* in experimental huts in Muheza, Tanzania

**DOI:** 10.1186/s12936-019-2973-x

**Published:** 2019-10-01

**Authors:** Eliningaya J. Kweka, Patrick K. Tungu, Aneth M. Mahande, Humphrey D. Mazigo, Subira Sayumwe, Shandala Msangi, Lucile Lyaruu, John Waweru, William Kisinza, James Wangai

**Affiliations:** 1 Department of Medical Parasitology and Entomology, School of Medicine, Catholic University of Health and Allied Sciences, P.O. Box 1464, Mwanza, Tanzania; 2 Mosquito Section, Division of Livestock and Human Health Disease Vector Control, Tropical Pesticides Research Institute, P.O. Box 3024, Arusha, Tanzania; 3 Amani Medical Research Centre, National Institute for Medical Research, P.O.Box 81, Muheza, Tanga Tanzania; 4 Division of Livestock and Human Health Disease Vector Control, Tropical Pesticides Research Institute, Mabogini Field Station, Moshi, Tanzania; 5 PestNet Kenya Ltd, P.O. BOX 51533-00200, Nairobi, Kenya

**Keywords:** *Anopheles funestus*, MAGNet, Experimental hut, Resistance, Exophily, Mortality, LLINs

## Abstract

**Background:**

The decline in malaria cases and vectors is major milestone in fighting against malaria. The efficacy of MAGNet long-lasting insecticidal nets (MAGNet LLIN), an alpha-cypermethrin incorporated long-lasting net, with the target dose ± 25% of 5.8 g active ingredient (AI)/kg (4.35–7.25 g AI/kg) was evaluated in six veranda-trap experimental huts in Muheza, Tanzania against freely flying wild population of *Anopheles funestus.*

**Methods:**

MAGNet LLINs were tested against wild, free-flying, host-seeking *An. funestus* mosquitoes over a period of 6 weeks (total of 36 nights in the huts). MAGNet LLIN efficacy was determined in terms of mosquito mortality, blood-feeding inhibition, deterrence, induced exiting, personal protection, and insecticidal killing over 20 washes according to WHO standardized procedures. Efficacy was compared with reference to a WHOPES recommended approved LLINs (DuraNet) and to a net conventionally treated (CTN) treated with alpha-cypermethrin at WHO-recommended dose and washed to just before cut-off point. The efficacy of MAGNet was evaluated in experimental huts against wild, free-flying, pyrethroid-resistant *An. funestus*. The WHO-susceptibility method was used to detect resistance in wild *Anopheles* exposed to 0.75% permethrin. Mosquito mortality, blood-feeding inhibition and personal protection were compared between untreated nets and standard LLINs. Blood-feeding rates were recorded and compared between the 20 times washed; blood-feeding rates between 20 times washed MAGNet LLIN and 20 times washed WHOPES-approved piperonyl butoxide (PBO)/pyrethroid were not statistically different (p > 0.05).

**Results:**

The results have evidently shown that MAGNet LLIN provides similar blood-feeding inhibition, exophily, mortality, and deterrence to the standard approved LLIN, thus meeting the WHOPES criteria for blood feeding. The significantly high feeding inhibition and personal protection over pyrethroid-resistant *An. funestus* recorded by both unwashed and 20 times washed MAGNet compared to the unwashed DuraNet, the WHOPES-approved standard pyrethroid-only LLIN provides proof of MAGNet meeting Phase II WHOPES criteria for a LLIN.

**Conclusion:**

Based on this study, MAGNet has been shown to have a promising impact on protection when 20 times washed against a highly resistant population of *An. funestus.*

## Background

Malaria vector control strategies have been a pillar to the success of malaria control globally [1]. African countries’ National Malaria Control Programmes (NMCP) have made substantial progress in malaria reduction through facilitation of free or subsidized long-lasting insecticidal bed net (LLIN) universal coverage campaigns with the aid of international donor agencies and governments for populations at malaria risk [2]. Although malaria vector populations across Africa have been reported to be declining [1, 3–5], malaria transmission is still high and concentrated in 10 countries, including Burkina Faso, Cameroon, Democratic Republic of the Congo, Ghana, Mali, Mozambique, Niger, Nigeria, Uganda, and United Republic of Tanzania) [1, 6]. The main tools that brought about malaria success to date are LLINs, indoor residual spraying (IRS) and appropriate diagnosis with drug prescriptions [1, 6]. LLINs are the most effective and feasible means of preventing malaria transmission in sub-Saharan Africa with a physical and chemical barrier [7–10]. With good LLIN technology, insecticidal efficacy can be maintained against anopheline mosquitoes for at least 3 years without need for further retreatment [8–11]. The demand for LLINs has attracted the interest of several pesticide companies to produce new brands of LLINs [1, 12, 13]. It is a pre-requisite for any new LLIN to be used by the community to pass a series of evaluation stages prior to its interim or full approval by WHOPES. WHO interim approval is given to a LLIN after it has successfully passed Phases I and II WHOPES evaluations, while the full approval is given after it has successfully passed Phase III evaluations [7–10, 12, 14].

This study assessed the bio-efficacy of unwashed and washed MAGNet LLINs: alpha-cypermethrin incorporated LLIN against wild, free-flying, pyrethroid-resistant *Anopheles funestus* field populations in northeastern Tanzania.

## Methods

### Description of the site and design of the trial

The experimental huts are located at a field site of the Amani Medical Research Centre in Zeneti village, located 30 km from Muheza District, northeastern Tanzania, between 5°13′24″S and 38°39′96″, at an altitude of 192.9 m above sea level. The area around Muheza is characterized with high malaria prevalence caused mainly by *Plasmodium falciparum* which is transmitted by *Anopheles gambiae* sensu stricto (s.s.) during the rainy season, and by *An. funestus* during the dry season [15, 16]. The area has a typical entomological inoculation rates (EIRs) of 34–405 infective bites per person per year [17]. *Anopheles gambiae* s.s. is the predominant vector in the wet season while *An. funestus* is predominant in the dry season [15, 17]. The huts are made to a standard traditional East African veranda trap-hut design, with brick walls plastered with mud on the inside, a wooden ceiling lined with hessian sackcloth, corrugated iron roof, open eaves, with window traps and veranda traps on each side. The huts are built on concrete plinths and surrounded by a water-filled moat to deter entry of scavenging ants. There are two screened veranda traps on opposite sides of the huts to capture any mosquitoes that exit via the open eaves (unbaffled). The eaves of the two open verandas are baffled inwardly to funnel host-seeking mosquitoes into the hut and to deter exiting through these openings. With this modified hut design there is no need to make any correction for escaping mosquitoes [18, 19].

### Description of the test product

The test product, MAGNet, is a candidate LLIN containing alpha-cypermethrin with the target dose ± 25% of 5.8 g active ingredient (AI)/kg (4.35–7.25 g AI/kg) incorporated into polyethylene, produced by VKA Polymers Co, India. Comparison of MAGNet was done against DuraNet, which is a WHOPES-recommended alpha-cypermethrin LLIN and against a negative control untreated net. This evaluation trial was undertaken in Muheza, Tanzania in six experimental huts which simulate domestic habitations, following closely WHO guidelines for laboratory and field testing of LLINs [20]. The LLINs were tested against free-flying, wild *An. funestus* s.s., a species that has a high frequency of pyrethroid resistance. Efficacy was expressed in terms of deterrence, induced exiting, mortality, blood-feeding inhibition, personal protection, and mass killing effect.

### Net preparation

The protocol developed by WHO was adopted for standard washing of LLINs for Phase II trial, over a 30-day period (i.e., by applying the regeneration time value that was established under Phase I of 24 h) [20, 21]. Nets were washed in aluminum bowls containing 10 L of dechlorinated water and 2 g/L soap (*Savon de Marseille*) using manual agitation. For each wash, nets were agitated for 3 min, left to soak for 4 min, and re-agitated for 3 min, for a total of 6-min agitation during a 10-min washing and soaking time. Agitation was done by stirring the net with a pole at 20 rotations per min. Rinsing was done twice using clean water (10 L per rinsing). Nets were dried horizontally in the shade then stored at ambient temperature between washes.

### Cone bioassays

The first bioassays were conducted using six nets each from each arm before the first wash. The wash was done immediately, and second round of bioassays were done after the wash. The second bioassays were conducted when all washings were completed and for a third time at the end of the hut experiments. Cone bioassays were conducted according to WHO procedures for cone tests [21]. A total of five non-blood-fed females of *An. gambiae*-susceptible Kisumu strain from Tropical Pesticides Research Institute (TPRI) insectaries were introduced in a cone and exposed for 3 min. Each net had five cones placed on five sides of the net (roof and four sides). Each side of the net had five replicates with a total of 25 mosquitoes per net side (i.e., 125 mosquitoes were used per net). The post-exposure knockdown was recorded after 60 min and mortality was scored after 24 h of exposure. In 24 h of monitoring, mosquitoes were provided with 10% sugar solution.

### Treatment arms and experimental hut trials

The following six treatment arms were compared:

1. Unwashed MAGNet.
2. Unwashed DuraNet.
3. MAGNet washed 20 times.
4. DuraNet washed 20 times.
5. Unwashed Interceptor.
6. Untreated polyester net.

The treatment arms were rotated through the huts according to a Latin square design. Data were collected for 36 nights. Three nets were available per treatment arm and each net was tested for three nights in each hut during the rotation. At the end of the rotation, the huts were cleaned and aired for 1 day and the treatments moved to the next hut.

Each net was deliberately holed with six holes (4 cm × 4 cm) to simulate a torn net. Sleepers slept in each hut once per week according to a strict rotation. Mosquitoes were collected from the floor, walls, exit traps and inside the nets, and scored as dead or alive and as fed or unfed. Live mosquitoes were held for 24 h to determine delayed mortality.

### Evaluation primary outcomes

1. Deterrence (reduction in hut entry relative to the control huts fitted with untreated nets);
2. Induced exiting (the proportion of mosquitoes that are found in exit traps and verandahs relative to control);
3. Blood-feeding inhibition (the reduction in blood feeding relative to the control);
4. Mortality (the proportion of mosquitoes killed relative to control).
5. Personal protection, which can be estimated by the calculation:
  - % personal protection = 100 (Bu − Bt)/Bu, where Bu = is the total number blood-fed in the huts with untreated nets, and Bt is the total number blood-fed in the huts with LLIN treated nets.
  - The overall killing effect of the treatment was estimated by the calculation: Insecticidal effect (%) = 100(Kt − Ku)/Tu, where Kt is the number killed in the huts with LLIN treated nets, Ku is the number dying in the huts with untreated nets, and Tu is the total collected from the huts with untreated nets.

### WHO insecticide susceptibility tests

The susceptibility tests were carried out using WHO test kits for adult mosquitoes [22]. Test papers impregnated with WHO-recommended discriminating dosage of 0.75% permethrin; papers were used as alternative pyrethroid because alpha-cypermethrin test papers were not available. The quality of the test papers was checked against a laboratory susceptible *An. gambiae* s.s. Kisumu strain before the actual testing started. Wild mosquitoes used in this test were F1 adults *An. funestus* and *An. gambiae* collected from the untreated experimental huts during and just after this trial. For each test, batches of 15–20 adult females were aspirated from paper cups and transferred into the holding tubes where they were held for 1 h before testing in exposure tubes lined with the test papers. Mosquitoes were exposed for 1 h and the number of mosquitoes knocked-down was recorded after 1 h. At the end of exposure period mosquitoes were transferred into holding tubes (lined with untreated papers) and provided with cotton pad soaked in 10% sugar placed on top of the holding tube. The mortality was scored 24 h post-exposure and each test was replicated depending on the number of mosquitoes collected.

### Data analysis

The main analyses were carried out using logistic regression for proportional data (adjusting for the effect of hut and sleeper) and Poisson regression for numeric data. Variance estimates were adjusted for clustering by each hut night of collection. The primary criteria in the evaluation were blood-feeding inhibition and mortality rates. The candidate LLIN meets the WHOPES Phase II efficacy criteria if it performs as well as or better than the reference LLIN when washed 20 times, in terms of blood-feeding inhibition and mortality rates. During analysis, *Culex quinquefasciatus* and *An. gambiae* sensu lato (s.l.) were dropped due to low density.

## Results

### Susceptibility test of *Anopheles funestus* from untreated huts

WHO susceptibility tests, on F1 adult *An. funestus* collected from the experimental huts with untreated nets and tested with permethrin papers, recorded mortality rates of 44%, indicating that *An. funestus* was resistant to pyrethroids (Table 1). Susceptibility tests on F1 *An. gambiae* collected from untreated huts recorded percentage mortality of 27% to permethrin (Table 2). Alpha-cypermethrin treated papers were not available.

**Table 1:** Experimental huts results against Zenet wild free flying *An. funestus* (number entering, proportions deterred, exiting, blood feeding, blood feeding inhibition and personal protection)

	Untreated net	MagNet LN	DuraNet LN	MagNet LN	DuraNet LN	Interceptor
Number of washes	0	Unwashed	Unwashed	20	20	Unwashed
Total females caught	182	202	288	180	190	171
Geometric mean females caught/night (95% C.I.)	3.4 (0.4–6.4)	3.8 (0.6–7)	6.2 (2.7–8.7)	4.1 (1.8–6.3)	3.7 (0.5–6.9)	3.5 (0.5–6.5)
% deterrence	–^a^	0^a^	0^a^	1.1^a^	0^a^	6^a^
Total females in verandah and exit traps	72	144	220	134	149	105
% exophily (95% C.I.)	39.6^a^ (32.5–46.7)	71.3^b^ (65.1–77.5)	76.4^b^ (71.5–81.3)	74.4^b^ (68.1–80.8)	78.4^b^ (72.6–84.3)	61.4^e^ (54.1–68.7)
Total females blood fed	36	45	34	15	28	43
% blood fed (95% C.I.)	19.8^abc^ (14–25.6)	22.3^b^ (16.5–28)	11.8^c^ (8.1–15.5)	8.3^d^ (4.3–12.4)	14.7^abc^ (9.7–19.8)	25.1^ab^ (18.6–31.6)
% blood feeding inhibition	–	0.0	40.4	58.1	2.6	0
% personal protection	–^a^	0^a^	5.5^a^	58.3^b^	22.2^ab^	0^a^
Total females died	12	12	12	11	21	14
% mortality (95% C.I.)	6.6^a^ (3–10.2)	5.9^a^ (2.7–9.2)	4.2^a^ (1.9–6.5)	6.1^a^ (2.6–9.6)	11.1^a^ (6.6–15.5)	8.2^a^ (4.1–12.3)
% mortality corrected for control	–^a^	0^a^	0^a^	0^a^	4.8^a^ (0–9.5)	1.7^a^ (0–5.7)
% overall killing effect	–^a^	0^a^	0^a^	0^a^	4.9^a^	1^a^

Numbers in the same row sharing a letter superscript do not differ significantly (p > 0.05)

**Table 2:** WHO susceptibility test with 0.75% permethrin test papers

	*Anopheles gambiae* (Kisumu)	*Anopheles gambiae* (Zenet collected)	*Anopheles funestus* (Zenet collected)
	0.05	0.05	0.05
Total females tested	100	68	64
% Mortality (95% C.I.)	100 (100–100)	27 (16.4–37.5)	44 (31.8–56.2)

### Number of mosquitoes collected from huts

*Anopheles funestus* were more abundant than *An. gambiae* during the trial. The average number of *An. funestus* per hut per night varied between 3 and 6 (Table 2). Insecticide-induced deterrence was not apparent with all treated arms.

### Exiting rates

All treated arms recorded significantly higher *An. funestus* exiting rates compared to that recorded by the untreated control arm. Furthermore, with exception, unwashed Interceptor LLIN exophily rates recorded by treated arms were statistically similar (p > 0.05) (Table 2).

### Blood-feeding

Blood feeding rates recorded by MAGNet washed 20 times (8.3%) was significantly lower (p > 0.05) than both unwashed and 20 times-washed DuraNet (11.8 and 14.7%, respectively), meaning that MAGNet washed 20 times is more protective than DuraNet, which is the WHO-approved LLIN (Table 2). Likewise, blood-feeding rate recorded by MAGNet after being washed 20 times was significantly lower (p < 0.05) than that of unwashed Interceptor LLIN, meaning that MAGNet washed 20 times is more protective than the WHO-approved unwashed Interceptor LLIN. The personal protection recorded by the 20 times-washed MAGNet (58.3%) was significantly higher than that recorded by all treatment arms (p > 0.05).

### Mortality

**Fig. 1:**
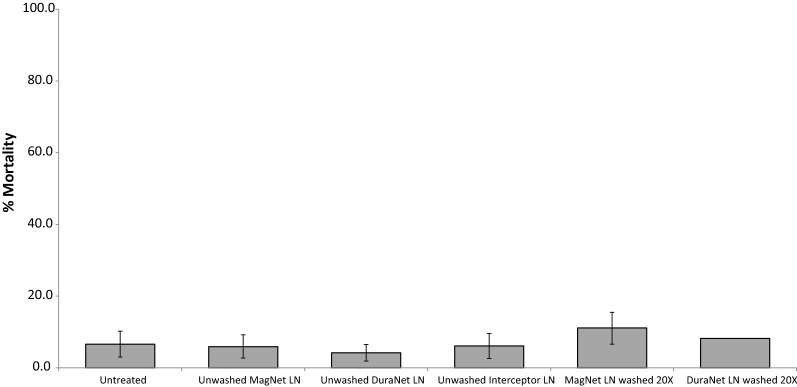
Experimental hut results. Mortality rates of wild, free-flying *An. funestus* in huts with different types of washed and unwashed nets

Mortalities of *An. funestus* recorded by all treated arms never reached above 11% (ranged between 4 and 11%). Although there were some differences in mortality rates among treated arms, the rates recorded by all treated arms and the untreated control were not statistically different (*p *> 0.05). Furthermore, mortality rates recorded by 20 times-washed MAGNet (6.1%) was similar statistically (p > 0.05) to that recorded by 20 times-washed WHO-approved DuraNet (11.1%) (Table 3, Fig. 1). Although there were differences in insecticide killing effect recorded between treated nets, the differences were not statistically significant (p > 0.05).

**Table 3:** Experimental huts results: %mortality and killing effect of *An. funestus*

	Untreated Net	MagNet LN	DuraNet LN	MagNet LN	DuraNet LN	Interceptor
Number of washes	0	Unwashed	Unwashed	20	20	Unwashed
Total females caught	182	202	288	180	190	171
Total females died	12	12	12	11	21	14
% mortality corrected for control (95% CI)	–^a^	0^a^	0^a^	0^a^	4.8^a^ (0.0–9.5)	1.7^a^ (0.0–5.7)
% overall killing effect	–^a^	0^a^	0^a^	0^a^	4.9^a^ (0.0–9.2)	1^a^

Percentage mortality and 95% CIs are back-transformed from values calculated by the blocked logistic regression model

Within a row, treatments not sharing a superscript letter differ significantly by blocked logistic regression (p < 0.05)

### Cone bioassay tests

The cone bioassays for all brands of nets used from Phase I and experimental huts had variations in knockdown effect and 24 h mortality rates as well (Table 4).

**Table 4:** Knockdown effect and 24 h mortality of unwashed and washed net brands evaluated

Treatment	Before washing			After washing, before hut trial			After hut trial		
	Number of mosquitoes tested	% knockdown (60 min)	% mortality (24 h)	Number of mosquitoes tested	% knockdown (60 min)	% mortality (24 h)	Number of mosquitoes tested	% knockdown (60 min)	% mortality (24 h)
Untreated polysterine nets	125	0.0	0.0	125	0.0	0.0	125	0.0	0.0
Unwashed MAGNet	125	90.4	100.0				125	100.0	100.0
MAGNet 20×	125	99.2	100.0	125	98.4	100.0	125	96.0	99.2
Unwashed DuraNet LN	125	100.0	100.0				125	97.6	98.4
DuraNet LN 20×	125	100.0	100.0	125	92.0	100.0	125	98.4	98.4
Unwashed Interceptor LN	125	95.2	100.0				125	96.8	98.4

## Discussion

The current study found the wild population composed *An. funestus* (95.1%) with *An. gambiae* s.l. (0.7%), which were not acceptable to be included in analysis, and 4.2% was made up by *Cx. quinquefasciatus*. This species composition of higher proportion of *An. funestus* is similar to that observed by previous studies conducted in Muheza, in Zeneth experimental huts [23–25]. In previous studies done in M’bé, Côte d’Ivoire it was found that the main mosquitoes entering a hut were *An. gambiae* s.l., which might be attributed to the ecological differences with Muheza influencing species composition variations [26].

Both unwashed DuraNet and MAGNet, and 20 times-washed DuraNet were found to have the lowest deterrence, while the highest was found to be Intercept unwashed (6%). The low deterrence rate observed in this current study was similar to that observed in another study based on PermaNet 3.0 unwashed and DuraNet washed 20 times against wild population of *An. gambiae* s.l. in Magugu, Moshi and Muheza [7, 8]. Other studies conducted in M’bé, Côte d’Ivoire showed the deterrence with MAGNet washed and unwashed to be higher than in control [26], while a study in India with MAGNet washed 25 times was not statistically different from other nets when compared in deterring mosquitoes from the huts [27]. These variations from one country to another might be attributed to the level of insecticide resistance among the wild mosquito populations in each locality.

In this study, the blood-feeding inhibition ranged between 0 and 58.1% for MAGNet unwashed and washed, respectively, compared to control. In a previous study conducted in Umbugweland using DuraNet, DuraNet had protection efficiency of 96.0 to 98.3% for 20 times washed and unwashed nets, respectively [8]. The huge variation in protection feeding inhibition might be due to high resistance level among wild population of *An. funestus* in Zeneth Muheza [28, 29]. This was contrary to other nets which loses protection efficacy after 20 washes [29]. The positive control nets (DuraNet) had protective efficacy of 40.4% before washing, which dropped to 2.6% after 20 washes. This sharp decline on protection efficacy was contrary to the previous study [8]. The data gathered from India showed that MAGNet had the highest blood-feeding inhibition, ranging from 43.3 to 48.1%, which was comparatively better than that in Muheza [27]; the study conducted in M’bé, Côte d’Ivoire had feeding inhibition of 40% for MAGNet which is lower than the Tanzania and India sites [26]. The blood-feeding increases of MAGNet from 0.0 to 58.1% for unwashed to 20 times washed in areas with resistant wild populations of mosquitoes has shown a promising result adding value to mosquito control toolbox for vector control.

In the assessment of the knockdown effect in laboratory before washing, after 20 washes, and after experimental hut trial, the knockdown shown was above the accepted WHO cut-off point: 90.4 to 99.2% [22]. These results are similar to that found in India and M’bé, Côte d’Ivoire with MAGNet [26, 27]. The trend observed in this study was similar to a previous trend observed for wild population in wild mosquitoes resistant to pyrethroids, organophosphates and carbamates [30–32]. In these trials, the mortality effect observed varied between mosquitoes collected in experimental huts with unwashed nets from 4.2 to 8.2%, while huts with 20 times washed nets varied from 6.1 to 11.1%. The mortality found in M’bé was 14–30% while in India it was 100% before and after experimental hut evaluations with similar LLIN brands [26, 27]. The observed mortality during trial was lower than recorded by other experimental hut LLIN screening in a similar study area [11, 23, 25]. The low mortality of wild population of mosquitoes observed in the Muheza study site is suggested to be attributed to high resistance frequency observed in wild populations in previous findings [33, 34]. Due to the main effect of resistance, the most important measures of evaluated LLIN strength was recorded in terms of personal protection and killing effect outcomes [8, 35].

The findings of this current study have shown MAGNet LN to have exophily rate of 71.3% when unwashed and 74.4% after 20 washes. These exophily results for unwashed and washed MAGNets has a similar trend to that found in India and M’bé, Côte d’Ivoire in MAGNet studies [26, 27]. The recorded exit rate in this study was found to be lower than that recorded in for the positive control nets. But still the exit rates recorded with the positive control (DuraNet) were less that those recorded with the same positive control in India and Tanzania, which was above 85% [8, 36]. This might be attributed to the variance in degree of insecticide resistance in the study area. Over the duration of this study there was no adverse effects reported by hut sleepers where MAGNets were used.

The current findings have shown that MAGNet is comparable to the registered standard nets (DuraNet and Interceptor) when 20 times washed or unwashed when tested against wild population of *An. funestus* or *An. gambiae* s.l. [8, 11, 36]. These results have revealed that the MAGNet, similarly to DuraNet, can be used in communities as physical and chemical barriers against malaria vectors. This concurs with studies conducted in the Solomon Islands and Tanzania, which have shown that community acceptance of LLIN usage was 68.7%, which can play a vital role in malaria transmission and vector decline [8, 37]. The higher response of acceptability for the nets used in the trial, including MAGNet, was found to be 71.1%. The highest acceptability is similar to previous studies in India and M’bé, Côte d’Ivoire using LLINs.

## Conclusion

Based on this study’s findings, MAGNet LLINs have been shown to have a promising impact on protection when 20 times washed than unwashed, with highly resistant populations of *An. funestus.* This study has given a new tool to complement existing tools in fighting malaria in areas with a high resistance vector population.

## Data Availability

All data associated with this manuscript conclusion have been presented in this paper.

## Abbreviations

CTN: conventionally treated nets
F1: first filio generation
LLINs: long-lasting insecticidal nets
NMCPs: National Malaria Control Programmes
TPRI: Tropical Pesticides Research Institute
WHO: World Health Organization
WHOPES: World Health Organization Pesticides Scheme
